# Impact of Air Pollution in Airway Diseases: Role of the Epithelial Cells (Cell Models and Biomarkers)

**DOI:** 10.3390/ijms23052799

**Published:** 2022-03-03

**Authors:** Giusy Daniela Albano, Angela Marina Montalbano, Rosalia Gagliardo, Giulia Anzalone, Mirella Profita

**Affiliations:** 1 Institute of Translational Pharmacology, National Research Council of Italy (CNR), 00133 Rome, Italy; giusydaniela.albano@cnr.it (G.D.A.); angelamarina.montalbano@cnr.it (A.M.M.); rosalia.gagliardo@cnr.it (R.G.); 2 Institute for Biomedical Research and Innovation (IRIB), National Research Council of Italy (CNR), 90100 Palermo, Italy; giulia.anzalone@cnr.it

**Keywords:** environmental pollution, airway diseases, epithelial cells, system biology, exposome

## Abstract

Biomedical research is multidisciplinary and often uses integrated approaches performing different experimental models with complementary functions. This approach is important to understand the pathogenetic mechanisms concerning the effects of environmental pollution on human health. The biological activity of the substances is investigated at least to three levels using molecular, cellular, and human tissue models. Each of these is able to give specific answers to experimental problems. A scientific approach, using biological methods (wet lab), cell cultures (cell lines or primary), isolated organs (three-dimensional cell cultures of primary epithelial cells), and animal organisms, including the human body, aimed to understand the effects of air pollution on the onset of diseases of the respiratory system. Biological methods are divided into three complementary models: in vitro, ex vivo, and in vivo. In vitro experiments do not require the use of whole organisms (in vivo study), while ex vivo experiments use isolated organs or parts of organs. The concept of complementarity and the informatic support are useful tools to organize, analyze, and interpret experimental data, with the aim of discussing scientific notions with objectivity and rationality in biology and medicine. In this scenario, the integrated and complementary use of different experimental models is important to obtain useful and global information that allows us to identify the effect of inhaled pollutants on the incidence of respiratory diseases in the exposed population. In this review, we focused our attention on the impact of air pollution in airway diseases with a rapid and descriptive analysis on the role of epithelium and on the experimental cell models useful to study the effect of toxicants on epithelial cells.

## 1. Environmental Pollution and Health of Respiratory System

There are two main types of air pollution: pollution of the external environments (outdoor) and pollution of the domestic environments (indoor) [1]. Today, air pollution is a current problem, since it can cause damage to the environment in which we live and to human health [2]. Air is a mixture of fundamental components (78% nitrogen and 21% oxygen) and secondary components (1% carbon dioxide and other gases). Environmental pollution is due to the presence of elements in the air deriving from human activities (industrial sources, domestic heating, vehicular traffic) and from natural phenomena (e.g., volcanic eruption). These last natural sources can produce elements potentially harmful for the human respiratory system.

People are exposed to contaminants through the respiratory tract and skin; they first reach the bloodstream and, subsequently, the organs, causing more or less serious damage to health [3,4,5]. Thus, the effects of atmospheric pollution affect the respiratory tract with acute symptoms and the circulatory system with cardiovascular events, leading to hospitalizations and mortality. In addition to the acute effects, long-term effects can also be had, including an alteration of lung function in adults, children, and adolescents. Specifically, in children and adolescents, chronic exposure to air pollution is associated with a reduction in forced vital capacity (FVC), which correlates with age and can be interpreted as a reduction in the lung growth and respiratory function of the lower airways [6,7]. Children, together with elderly persons, are the most sensitive subjects to environmental pollution; to these are added subjects with chronic respiratory diseases such as asthma and chronic obstructive pulmonary disease (COPD).

The substances that modify the composition of atmospheric air, causing pathological alterations for the respiratory system, are numerous and of different nature. In the recent decades, the number of subjects with chronic respiratory diseases increased exponentially in relation to air pollution [8,9,10,11]. Already in the 1960s, when the main source of air pollution (outdoor) in cities was the combustion of coal, a close association between urban pollution and symptoms of bronchitis was observed, which was generally accompanied by decreased respiratory functions [12]. An analysis from the *Global Burden of Diseases Study* stated that air pollution is the second most common cause of death and disability for people with COPD [13].

Air pollutants are largely produced by vehicle exhausts and are represented by carbon monoxide (CO), nitrogen oxides (NOx), sulfur dioxide (SO_2_), polycyclic aromatic hydrocarbons (PAH), particulate matter (PM) and suspended powders, cadmium [8], ozone, and volatile organic compounds (VOCs), which include benzene, toluene, and xylene, among others. Note that environmental air pollutants also include heavy metals such as lead, aluminum, and mercury [9]. It is worth particular attention that carbon monoxide (CO), once inhaled, binds to hemoglobin, a protein of red blood cells responsible for transporting oxygen, forming carboxyhemoglobin (COHb). This bond is much more stable (about 200–300 times) than hemoglobin and oxygen; in this way, the CO prevents the normal transport of oxygen to the tissues, thus creating toxicological effects of different magnitudes [14]. In fact, recent studies have reported that short-term exposure to environmental CO determines the increased risk of developing respiratory diseases (bronchiectasis, pneumonia, and asthma) [15]. As for nitrogen oxides (NOx), they are compounds of both natural origin (volcanic eruptions and soil emissions) and anthropogenic, and their ability to form nitric acid in the mucosa of the airways and on the skin makes them toxic to humans and animals.

Short-term exposure to nitrogen dioxide (NO_2_), especially in subjects with pre-existing lung disease, causes an increase in the exacerbation and an increase in bronchial reactivity. In addition, some meta-analysis studies have reported an association between NO_2_ concentration and mortality from lung cancer [16] as well as respiratory and cardiovascular diseases [17]. Sulfur dioxide (SO_2_) also causes harmful effects on human health. Exposure to this substance for a short period result in a significant increase in hospital admissions for respiratory diseases; 10 out of 25 are associated with an increase in symptoms and a reduction in lung function, while long-term exposure causes premature death and impairs the function of the airways, causing heart problems [18,19].

Polycyclic Aromatic Hydrocarbons (PAHs) is a class of numerous organic compounds structurally characterized by the presence of two or more aromatic rings condensed together. PAHs include benzopyrene, acenaphthylene, anthracene, and fluoranthene, among others, and according to the Environmental Protection Agency, benzo-[a]-pyrene (B [a] P) is the most potent. PAHs are different for environmental sources and chemical characteristics. However, PAHs are formed during the incomplete combustion of organic products such as coal, oil, gases, or waste. They are present in the air as mixtures of many dozens of molecules, which are often present in different and variable proportions. The presence of PAH mixtures in the air makes it difficult to understand the specific mechanisms by which PAHs affect human health. These compounds are recognized as toxic, mutagenic, and carcinogenic and are an important risk factor for lung cancer [20]. At the same time, the International Cancer Research Agency (IARC) identifies lead, cadmium, nickel, and arsenic as the most representative heavy metals for environmental risk and, both for their massive use and for their toxicity, are classified as carcinogenic to humans. Among these, nickel can have negative effects on the respiratory system, while lead is absorbed by the pulmonary epithelium and enters the bloodstream and is deposited in various organs [21]. Therefore, the current legislation (Legislative Decree 155/2010) has defined a limit value for lead, arsenic, cadmium, and nickel in the air. On the other hand, fine powders (e.g., PM_10_ and PM_2.5_) are polluting particles present in the air, of organic or inorganic nature, capable of adsorbing on their surface various substances with toxic properties, such as sulfates, nitrates, metals, and volatile compounds.

The toxicity of these substances to the respiratory system increases as their size decreases. However, air contaminants are also present in the confined (indoor) environments where we live and work on a basis daily (formaldehyde, radon, volatile organic compounds, polycyclic hydrocarbons, etc.) [8], and numerous studies have documented a link between increased mortality cardiovascular or respiratory and exposure to fine particulate matter (PM_10_ and PM_2.5_) [22,23,24,25]. Furthermore, it has been shown that elevated concentrations of atmospheric PM are associated with an increase in hospital admissions for heart or respiratory diseases in subjects at risk (e.g., patients with asthma), and that chronic exposures to these substances are a risk factor for cancer [26,27]. Respirable “particulate matter” (PM) has been identified by the US Environmental Protection Agenc as a “criteria air pollutant” together with carbon monoxide, ground level ozone, nitrogen dioxide, sulfur dioxide, and lead [28,29,30].

In this regard, the European project ESCAPE (European study of cohorts for air pollution effects), which included nine European countries including Italy, highlighted the relationship between lung cancer, the degree of pollution in the areas of residence, and exposure to fine dust (PM_10_ and PM_2.5_). However, increases in lung cancer cases were also recorded in groups exposed to pollution levels below the maximum limits established under current European legislation (equal to 40 μg/m^3^ of PM_10_ and 25 μg/m^3^ of PM_2.5_) [31]. Therefore, the World Health Organization (WHO) defined that it is not possible to establish a threshold value below which PM_2.5_ is not harmful to people; therefore, the environmental concentration of PM_10_ and PM_2.5_ in the air should be kept as low as possible [32]. PM_10_, PM_2.5_, and nitrogen oxides (NOx) are among the main atmospheric pollutants considered persistent carcinogens. They are monitored at European levels; about 90% of city dwellers are exposed to concentrations of pollutants higher than the values considered harmful to health [33,34]. Furthermore, a recent study showed a close relationship between increased atmospheric concentrations of nitrogen dioxide and PM_2.5_, death rate, and hospital admissions for COPD [35], while lung cancer cases have been linked to the presence of PM in the deepest part of the lungs [36]. The composition of PM mixtures from underground railways is very different of PM mixtures from urban areas. PM mixtures from underground railways are rich in metals associated with wheel, track, and brake wear and electrical arc and component wear [37]. When particulate matter enters the respiratory tract, an interaction is created between lung epithelium and the immune system: this activates the local inflammatory response associated with the disease [38]. Epidemiological and experimental studies suggest that among particulate air pollutants, diesel exhaust particles seriously affect the increase in morbidity and mortality from respiratory diseases. In fact, in urban areas, fine particulate matter produced by diesel engines (diesel exhaust particle cells) is a major source of PM_2.5_ as it is readily inhaled deep into the lungs and remains there for a long time, resulting in cellular responses that generate intense inflammatory reactions in the airways [37].

Respiratory diseases are multifactorial and therefore have various risk factors, including active and passive smoking, air pollution [39], and cellular oxidative stress related to environmental contamination. This last is involved in the deregulation of cellular senescence and in severe airway disease with public health implications affecting the respiratory system [40]. In the sites characterized by the presence of large industrial settlements, refineries of petrochemical nature, or chemical plants, there are the atmospheric pollutants. The air pollution can affect the respiratory system, causing malignant tumors or chronic inflammatory diseases of the lung in both adult and asthmatic children with a consequent increase in hospitalizations for asthma at a young age [40].

Among the most dangerous compounds for human health, Persistent Organic Pollutants (POPs) should be mentioned, which are halogenated compounds persistent in the environment. This family of pollutants includes polychlorinated or polychlorinated biphenyls (PCBs), organochlorine pesticides, and polybrominated diphenyl ethers (PBDEs), which are all highly toxic. As for PBDEs, these are known as emerging contaminants generally referred to as “flame retardants”. According to the European Food Safety Authority (EFSA 2011), the main source of PBDE exposure is food of animal origin with high fat content (fish, meat, and dairy products) [41]. They are ubiquitous and lipophilic, and they tend to accumulate in adipose tissue and interfere with the immune system [9]. Therefore, humans are exposed to PBDEs through diet as well as through the accidental ingestion of dust, skin contact, and inhalation. Atmospheric levels of PBDEs depend on deposition processes, weather conditions, long-range atmospheric transport, and the proximity of PBDE sources to the sampling site (urban/industrial or background locations). A recent study monitored atmospheric PBDE levels (PBDE 28, 47, 85, 99, 100, 153, 154, 183, and 209) in Central Europe, and the results indicate a global increase in low-bromine PBDEs in atmosphere [42]. This effect is due to the photolysis process, which favors the de-bromination of PBDEs with a higher bromine content.

Emerging contaminants such as the flame retardants (polybromo-diphenil, PBDEs ethers) PBDE-47, -99, and -209 are widespread in indoor and outdoor environmental contamination. These substances are present in fabrics, electrical materials, dust; e.g., they induce pulmonary toxicity by promoting an inflammatory response in lung epithelial cells [43,44,45]. The same PBDE profile has been described in southern Europe by Besis et al. [46], while Pozo et al. identified, of 26 PBDEs routinely analyzed, the presence of three (PBDE-47, -99 and -100) in the coastal areas of Sicily [47]. These substances are highly carcinogenic, and they certainly interfere with the endocrine system (endocrine disruptors). They have a negative effect on the reproductive system and, in mouse study models, alter the immune system [48], reducing the reactivity of macrophages compromising the associated immune response to cellular signals regulated by the TLR4/NF-kB pathway. A further experimental study showed that congeners -47, -99, and -209 are transferred from the mother to the fetus via the placenta, representing a risk factor for the development and growth of the fetus [49], often leading to the development of child allergic/asthmatic phenotype [9]. Due to their presence in the environment and their proven toxicity, commercial blends of Penta and Octa-PBDE have been banned in the European Union (EU) and some US states since 2004 [50] and in Canada since 2006. In 2009, they were designated as new persistent organic pollutants (POPs), and the Stockholm Convention banned their production and established the decrease in the use of commercial blends with more than seven bromine atoms (UNEP, 2015). Furthermore, in 2008, the EU banned the use of Deca-PBDE in electronic and electrical applications at concentrations higher than 0.1% of the total concentration of PBDEs [51]. Finally, despite these measures, there are still few studies to date that seek to identify scientific evidence showing a potential relationship between the effects of inhalation of these substances and chronic inflammatory lung diseases.

## 2. Role of Bronchial Epithelial Cells in Respiratory Diseases

The pulmonary epithelium separates the air introduced in the lungs from the underlying aqueous interstitial compartment. It acts as a barrier for defense against a wide range of inhalation stimuli ranging from pathogens present in the air to toxins and particulates [52]. It has different defense mechanisms such as mucociliary clearance, ion secretions, the production of anti-inflammatory substances, i.e., antibacterial and antioxidant molecules in the mucus, providing an immune defense system organ [53]. The epithelium of the airways plays a key role in orchestrating cellular mechanisms involved in the regulation of the innate and adaptive immune response. It takes part in the inflammatory response and remodeling phenomena of lung tissue during respiratory diseases [54]. Inhaled harmful substances (exogenous noxae) and environmental contaminants reach the bronchi and settle, exerting their cytotoxic activity in the lung epithelium [55]. In this way, the pulmonary epithelium, by regulating the balance of lung fluid and modulating the metabolism and clearance of inhalation of external agents, promotes the secretion of numerous mediators responsible for the recruitment and activation of cells inflammatory response to injury or infection in the underlying tissues [55].

The epithelium is a barrier between the environment and the organism. It is selective and allows only the passage of soluble molecules and ions from the paracellular spaces. In this manner, the epithelium prevents the migration of pathogens or pollutants from the lumen to the interstitium. This action is controlled mainly by the intercellular junctions: tight junctions, adherent junctions, and desmosomes [56]. The integrity of the airway epithelium is essential to ensure the functions of epithelial tissue and to regulate the lung inflammation. The consequent interruption of tension of intercellular junctions compromises the integrity of airway functions. These alterations underlie pathological conditions associated with pulmonary and cardiovascular diseases [57].

The pseudo-stratification of the airway epithelium is constituted to cells with different morphology and dimensions, leaning against the basement membrane. The human airway epithelial surface consists of bronchial compartment with ciliated cells, goblet cells (mucus production), and Clara cells, while the alveolar compartment is lined with pneumocytes: alveolar epithelial cells of type I (AT I) and alveolar epithelial cells of type II (AT II). AT I cells are terminally differentiated thin squamous cells that cover 90–95% of the alveolar space, while AT II cells are cuboidal cells that make up 15% of total lung cells [57].

Alveolar macrophages (AMs) are another important cell type in the lung compartment. This sub-population of tissue-resident cells is a constant cell pool essential for lung homeostasis [58,59]. AMs protect the alveolar space from foreign material engulfing and eliminating it. In this process, AMs play a central role in the regulatory mechanisms of the innate and adaptive immunity of the lung [60]. In fact, the mechanism of immunological response to the inhalation of environmental contaminants is associated to cell-to-cell interaction and cell communication between epithelial cells of the lung and AMs. In this context, extracellular vesicles exchange (exosomes, microvesicles, and apoptotic bodies) containing proteins, nucleic acids, and lipids plays a relevant role in the immunological communication [61]. In vitro/ex vivo studies obtained using cell models including co-cultures represent a useful tool to describe the immunological interaction between lung epithelial cells and monocytes/macrophages. Furthermore, they help to define the effects (endpoint) of toxicity from environmental contamination [62].

The environmental factors can alter the normal function of epithelial cells by promoting lung diseases such as chronic obstructive pulmonary disease (COPD), asthma, and lung cancer [63]. Following a response to inhaled noxae, the bronchial epithelial cells are a source of interleukin hyperproduction (IL-1α, IL-1β, IL-6, IL-8, and IL-18) and mediators such as tumor necrosis factor-α (TNF -α) [64]. Interleukins and mediators are involved in cell communication and in the activation of an inflammatory response in the lung [65,66,67,68]. Pro-inflammatory agents such as cationic peptides, cell protease receptors, and prostaglandins as well as non-proteolytic allergens, bacterial exotoxins, Damage-Associated Molecular Pattern (DAMP), and histamine are exogenous factors triggering the release of cytokines IL-6, IL-8, granulocyte-macrophage colony-stimulating factor (GM-CSF), and monocyte chemoattractant protein 1 (MCP-1) in human airway epithelia [38,68,69,70,71,72,73,74,75,76,77,78]. They alter mechanisms of cellular transport of calcium ion Ca+ in bronchial epithelial cells [39,79]. IL-25, IL-33, and thymic stromal lymphopoietin (TSLP) are mediators released by bronchial epithelial cells following viral, fungal, and bacterial infections [80,81,82,83,84,85,86,87,88]. After a continuous inhalation of stimuli, epithelial cells produce reactive oxygen species (ROS) as a consequence of an imbalance between cellular oxidants and antioxidants. ROS have a fundamental role in the mechanism of inflammatory processes and tissue damage of the lung. The increased release of mediators in the airways activates hyperplastic bronchial epithelial cells to a strong production of mucus that obstructs the lung lumen, damages cellular repair mechanisms, and promotes squamous metaplasia (multilayered epithelium). In this way, the increase in the deposition of extracellular matrix underlying the epithelium is favored, generating fibrosis and thickening of the wall of the airways. This phenomenon is named “remodeling of the airways”. It occurs in some pathological conditions of the respiratory system [89].

A balanced inflammatory response is a necessary requirement to successfully protect the lungs. An excessive inflammatory response favors damage to lung tissue with consequent alteration of functional parameters and of the physiological mechanisms of respiration at the basis of diseases of the respiratory system. Therefore, the evaluation of the response to environmental exogenous noxae becomes important for establishing how the epithelium intervenes in orchestrating and directing the immunological response, taking an active part in the functions of the individual’s immune system [53,54,90].

Thus, the respiratory epithelium constitutes an active part of the immune system. It responds to environmental stimuli and secretes mediators, causes damage to the mucosa, leads to a reduction in mucociliary clearance, promotes the oxidation of lipids, causes alteration of the membrane permeability, destroys the cytoskeletal components, and causes the loss of integrity of the epithelial layer. These cellular events dictated by the epithelium promote the recruitment of inflammatory cells (mast cells, macrophages, and dendritic cells). Once activated, the inflammatory cells reach the lung tissue, enhance local inflammation, and influence the increased production of mediators both by epithelial cells and by the endothelial cells of the airways. The result is an altered expression of cell adhesion molecules that favor the unregulated recruitment of infiltrating cells such as eosinophils, neutrophils, and lymphocytes [53,54,90]. A bronchial epithelium that increases *biomarker* release is an active epithelium and can be considered a sensor of environmental contamination.

The lung is constantly exposed to inhaled pathogens (bacteria, virus) and particulate matter (exhaust gas, diesel, wood smoke). The epithelium of the airways from the trachea to the alveoli plays a fundamental role in maintaining the normal function of the lung tissues [54]. Therefore, the identification of the cellular and molecular processes that regulate the development of epithelial tissue, its differentiation pathway, the recognition of pathogens, antimicrobial response and responses in general to inhaled noxae (pathogens, environmental contaminants), favors the understanding of the physiological dysfunctions that participate in the pathogenesis of diseases, compromising the health of the respiratory system [91,92,93].

## 3. Effects of Air Contaminants on Epithelial Cells of the Lung

The main pollutants monitored in the air are sulfur dioxide (SO_2_), nitrogen oxides (NO and NO_2_), ozone (O_3_), carbon monoxide (CO), benzene (C_6_H_6_), particulate matter PM_10_ (particles with aerodynamic diameter < 10 μm), particulate material PM_2.5_ (particles with aerodynamic diameter < 2.5 μm), benzo (a) pyrene (B (a) P), arsenic (As), cadmium (Cd), and nickel (Ni) [94]. Many epidemiological studies describe the relationship between these contaminants and diseases of the respiratory system [95,96].

Air pollution is mainly related to urban centers, industrial activities, and road traffic. Numerous epidemiological and in vitro studies linked air pollution to various harmful effects on human health. Atmospheric contaminants are a heterogeneous mixture of particles suspended in a liquid and gaseous phase. They are involved in the interruption of the redox homeostasis known as cellular oxidative stress in relation to the mechanisms of inflammation and cell death that involve phenomena of cell necrosis, apoptosis, or autophagy. The activation or repression of the apoptotic process, as an adaptive response to xenobiotics, can lead to acute or chronic toxicity; therefore, the oxidative stress induced by environmental pollutants plays a central role on cellular impacts ranging from cytoprotecting, to cytotoxicity, to apoptosis [97].

This paragraph will describe the effects of contaminants present in the air on cellular toxicity in the respiratory epithelium. PMs should be considered as a toxicologically heterogeneous class of chemicals rather than a single homogeneous entity. Much evidence shows a broad spectrum of adverse effects of PM on the airways and described different mechanisms by which these effects are exerted on lung epithelial cells. The airway epithelium is the main place of deposition of PM, and it plays a critical role in initiating the immunological response to these substances. Coarse PMs are deposited mainly in the upper airways, trapped by cilia and mucus, while the thinner PM penetrate more easily reaching the bronchioles and terminal alveoli [37]. The latter enter the circulation through the gas–blood barrier, persisting for several months after inhalation [97]. Negative respiratory outcomes associated with PM exposure are exacerbations of asthma and COPD, idiopathic pulmonary fibrosis, and lung cancer [98,99,100,101].

Oxidative stress occurs when there is an excess of potentially harmful oxidants, including free radicals and reactive oxygen species (ROS), which overcomes the antioxidant defenses of the cells. Consequently, there is oxidation of cellular components, such as nucleic acids, proteins, and lipids, which favor tissue lesions and the infiltration of inflammatory cells [102]. PM generate oxidative stress and induce antioxidant and inflammatory response, triggering alterations of epithelial cells activities compatible with the main causes of respiratory diseases. The capacity to generate ROS, and therefore to generate oxidative stress in bronchial epithelial cells, increases as the PM size decreases. PM can exert oxidative stress through several mechanisms [103]. Soluble components of PM, particularly transition metals, can generate ROS due to their ability to act as electron donors [104]. Transition metals can exist in multiple oxidation states and then donate electrons to molecular oxygen to generate ROS, forming superoxide, hydrogen peroxide, and hydroxyl radicals, as well as potentially damaging reactive nitrogen and sulfur species [104,105]. The exposure of epithelial cells to PM increases ROS production. The oxidase nicotinamide adenine dinucleotide phosphate (NADPH) and duox oxidase 1 (DUOX1) are key mediators of inflammatory effects of PM. The exposure to PM_2.5_ increases oxidative cell stress through the increased expression of duox oxidase 1 (DUOX1) in epithelial cells from human bronchi and DUOX1 and NADPH from both epithelial cells from the bronchial and alveolar levels [106,107,108]. Moreover, PMs induce mitochondrial toxicity with the consequent overproduction of mitochondrial ROS, deregulation of the electron transport, loss of mitochondrial membrane potential, and impaired oxidative phosphorylation in bronchial epithelial cells [109,110,111].

Therefore, the action of PM on the respiratory system is mainly associated with tissue damage, alterations in mucociliary clearance, production of cytokines, and activation of mitophagy mechanisms in epithelial cells. Their action is exerted through intracellular signals of the MAPK kinase and NF-κB pathway, and it promote oxidative stress mechanisms, cytokine synthesis (IL-1β, IL-6, and IL-8), as well as metallopeptidases-9 (MMP-9) and cyclooxygenase-2 (COX-2). All these markers are involved in the pathogenesis of respiratory diseases [112]. Exposure to PM_2.5_ also reduces the expression of miR-331 via the ROS/PI3K/Akt pathway resulting in sustained and prolonged activation through an increase in the expression of the IKK-β kinase, NF-κB pathway, which is involved in the regulation of pro-inflammatory cytokine transcription in human airway epithelial cells [113]. Furthermore, PMs through the involvement of receptors Toll-like receptors (TLRs), mainly TLR4 and TLR2, induce the translocation of NF-κB into the nucleus and influence the production of cytokines IL-6 and IL-8 [114].

The underground PMs increase the number of oxidized biomolecules and therefore generate greater damage to the DNA of epithelial cell lines A549 [115,116]. The study of the effect of underground PM has shown their greater ability to generate ROS with respect to PM from other sources (urban, road wear, diesel, and wood burning) [37]. Short-term exposure (1 h) to the particulate matter of diesel exhaust of healthy volunteers determines acute inflammatory responses in the lower respiratory tract, which is observed with biological evaluations obtained in broncho-alveolar lavage and in biopsies of the mucosa of these subjects [117].

A cellular defense mechanism against oxidative stress is the phenomenon of autophagy, which is a homeostatic aeration that reduces the cytoplasmic volume by degrading organelles and proteins damaged in the cell. Thus, a lysosome-dependent degradation process occurs, and new organelles and proteins are synthesized in substitution [118,119,120]. Previous studies demonstrated that the exposure to PM induces the formation of reactive oxygen species (ROS) and increases levels of autophagy and cell death [120,121]. In addition, recent in vivo and in vitro models have shown that oxidative damage caused by exposure to diesel can activate the antioxidant response and maintain cellular homeostasis due to the activity of nuclear transcription factor 2-like 2 (Nrf2) in bronchial epithelial cells [122]. The functional enrichment of differentially expressed genes indicates that exposure to diesel induces the activation of genes involved in TNFα production, via NF-kB, and promotes inflammatory response and hypoxia in bronchial epithelial cells. Exposure to diesel particulate matter also induces the secretion of inflammation biomarkers (CCXL2, EPGN, GREM1, IL1A, IL1B, IL6, IL24, EREG, VEGF) and transcription factors (NFE2L2, MAFF, HES1, FOSL1, TGIF1) involved in pulmonary cardiovascular diseases, involving epithelial tissue. In addition, four genes (STAT3, HIF1a, NFKB1, KRAS) have been identified as major regulators of the transcriptional response of bronchial epithelial cells exposed to diesel exhaust [123].

In vitro and ex vivo experimental studies identified carcinogenic effects of PAHs, which are often related to their ability to bind DNA. PAHs promote DNA cross-linking mechanisms, causing a series of cellular effects that trigger carcinogenesis in numerous cell types including lung epithelial cells. These substances cause cell toxicity, producing reactive oxygen species (ROS), and regulate the processes of cell death (apoptosis). In addition, cell toxicity and the weakening of the immune system by industrial pollutants PAH favors the uncontrolled cell proliferation and the progression toward the mechanisms of lung cancer [124].

After cigarette smoke inhalation, the lung can absorb large amounts of cadmium (Cd) or occupational exposure [125]. Cd causes the transformation of human bronchial epithelial cells and plays an important role in lung carcinogenesis [126,127]. Among the various mechanisms of Cd-induced malignant transformation, together with the upregulation of SATB2 transcription signal signals and the downregulation of methyltransferase MGMT, there are increased levels of oxidative stress phenomena in bronchial epithelial cells. Cd induces oxidative stress by depleting glutathione and protein-bound sulfhydryl groups, leading to an increase in the production of reactive oxygen species (ROS). It can also act as an epi-mutagen through the hypermethylation of gene promoters or by altering post-transcriptional modifications of histones [128]. Prolonged exposure to Cd promotes the malignant progression of lung cancer by activating the cell signaling pathway involving Notch1, along with HIF-1 and IGF-1R/Akt/ERK/S6K1 in epithelial cell lines [129].

**Figure 1:**
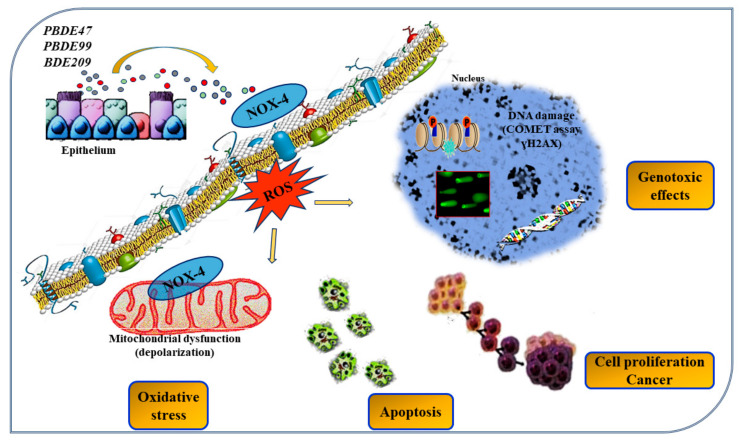
Effects of PBDE-47, -99, and -209 flame retardants in bronchial epithelial cells. PBDE-47, -99, and -209 cause DNA damage of epithelial cells and alter the activity of histone protein γ-H2AX in in vitro/ex vivo cell models of human bronchial epithelial cells.

PBDE causes the oxidative stress and cytotoxic mechanisms, mitochondrial damage, and DNA damage in various organs and tissues. PBDEs induce pulmonary toxicity, promoting an inflammatory response in lung epithelial cells [127,128,129]. Recently, Montalbano et al. show that PBDE-47, -99, and -209 cause DNA damage of epithelial cells and alter the activity of histone protein γ-H2AX in vitro/ex vivo cell model of human bronchial epithelial cells (Figure 1) [130]. Furthermore, Albano et al. demonstrate by in vitro/ex vivo studies obtained with a three-dimensional cell model of the “Air–liquid interface” that the inhalation of PBDE-47, -99, and -209 might compromise human lung health, promoting oxidative stress and increasing the expression of NADPH oxidase (NOX-4), interleukin IL-8 production, the loss of epithelial barrier integrity, reducing the transepithelial electrical resistance (TEER) and zonula occludens-1 (ZO-1) expression, the uncontrolled production of mucus, and the alteration of physicochemical and biological properties of fluids in bronchial epithelial tissues [131]. These results define the importance of studying, with biological systems in vitro/ex vivo, the still-unknown action of emerging contaminants introduced by inhalation into the airways. These methods can be used to understand the role of environmental pollutant in the pathogenesis of respiratory diseases and to identify biosensors of disease.

The toxicology of mixtures has recently generated greater interest than the evaluation of the effect of single substances. The toxicity of pollutant mixtures could represent a more accurate way to understand in *real time* the effects of environmental contamination on the respiratory system and to determine harmful effects relevant to the health of people. Therefore, the use of suitable cell models could create broad prospects for identifying the toxicology of mixtures.

## 4. Cell Systems to Study the Effects of Environmental Contaminants in Respiratory Diseases

One of the “gold standards” of environmental scientific research is to obtain data to understand the effects of exposure to inhaled toxic substances on human health. In vivo *studies* were used only to collect data related to an indirect effect of pollutants. These studied do not establish a direct relationship between ethical and safety precautions, high costs, very long periods, and environment with pathogenetic alterations regarding human health. Furthermore, data obtained from observational studies of subjects in the areas of environmental contamination are to lower the resolution of pathological effects at the cellular and molecular levels [132].

For many years, scientific research has used animal models as the main tools to evaluate the effects of inhaled substances on human health. However, results obtained in mouse models are not always able to predict diseases, and their general use for research purposes has raised growing public and animal welfare concerns. The scientific research pushes toward the use of alternative and innovative in vitro/ex vivo experimental models [133,134,135,136]. The history of experimental models began in 1885 with the zoologist Wilhelm Roux. He was the pioneer of experimental embryology studying the embryonic chicken cells in saline for several days. However, only in the mid-1950s, Harry Eagle gave a significant boost to this area of research by studying and identifying the nutrients needed by cells in culture [137,138]. To date, the cell cultures and in vitro/ex vivo models are essential for the identification and the study of the effects of inhaled *noxae*, which are represented by atmospheric pollutants, on biological systems [139]. In recent decades, tissue engineering approaches have made enormous progress, and several in vitro models have been established to study the effect of inhalation toxicity and disease. The aims of these studies are to improve the understanding of pathophysiological processes and to provide new and more independent experimental systems for pharmacological and toxicological studies.

In vitro lung models are currently available for all important segments of the respiratory system starting from the nasal cavity and trachea to the proximal and distal airways [140]. Traditional two-dimensional (2D) monolayer culture models (also called “submerged cultures”) are used to identify molecules involved in the signaling of altered cellular and molecular mechanisms that arise in the case of pulmonary toxicity. However, these models lack the key features of the human airway microenvironment that are essential for accurately studying the toxic effects of inhaled exogenous *noxae*.

**Figure 2:**
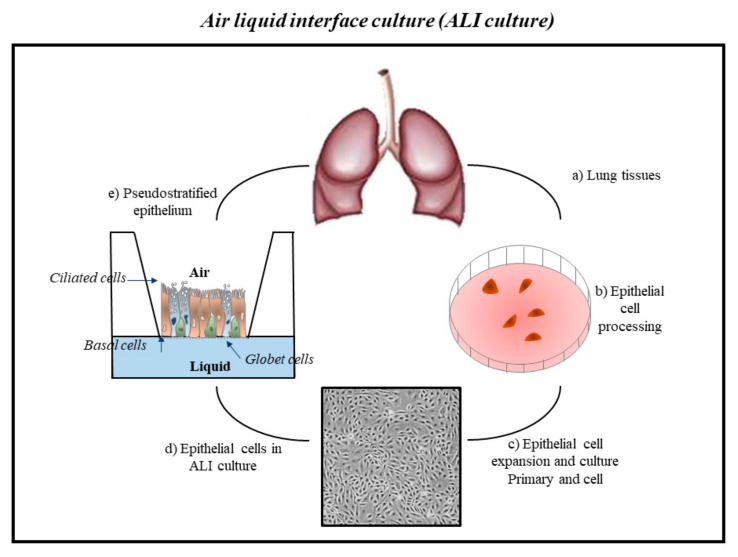
Three-dimensional (3D) ALI cultures of epithelial cells (primary and cell line) to study the effects of environmental contaminants in airway disease. Steps to obtain primary epithelial cells from human tissue: (**a**) Collection of bronchial biopsies or surgical specimens; (**b**) epithelial cell processing: tissue was dissociated, resuspended in bronchial epithelial growth medium; (**c**) cell expansion and culture of epithelial cells; (**d**) the cells are seeded onto the microporous membrane pre-coated with collagen in submerged conditions until confluence and culture in ALI; (**e**) reaching the confluence, the cells begin to lift at the air–liquid interface starting the differentiation fed by the culture medium in the basolateral side. Hence, the epithelial cells differentiate in the pseudostratified phenotype and build a tissue such as the epithelium of the lung.

Recently, the traditional method of two-dimensional single-layer cell culture was replaced with more innovative methods, since it does not faithfully reflect what is observed within tissues in vivo [141,142]. The main reason for the inadequacy of these cell culture systems is the lack of the architectural support and heterogeneity typical of the cells of the lung tissue. This awareness has led to an increase in the development of more complex three-dimensional (3D) models in which cells can grow in multiple directions in order to better reflect the cellular interaction present in the natural environment in vivo. Furthermore, multiple cell types may be present in these models [44,143,144,145,146] and an extracellular matrix, thus allowing to mimic a model similar to one in vivo. The “*air–liquid interface*” (ALI) culture is an example of these innovative models of cell culture (Figure 2).

This biological system of epithelial cells has a well-differentiated epithelium similar to human airways. Thus, epithelial cell cultured in ALI represents a valuable tool for scientific research to study the toxic effect of inhaled chemicals on the human health affecting respiratory diseases, providing the opportunity to evaluate and identify important cellular and molecular mechanisms [147].

**Figure 3:**
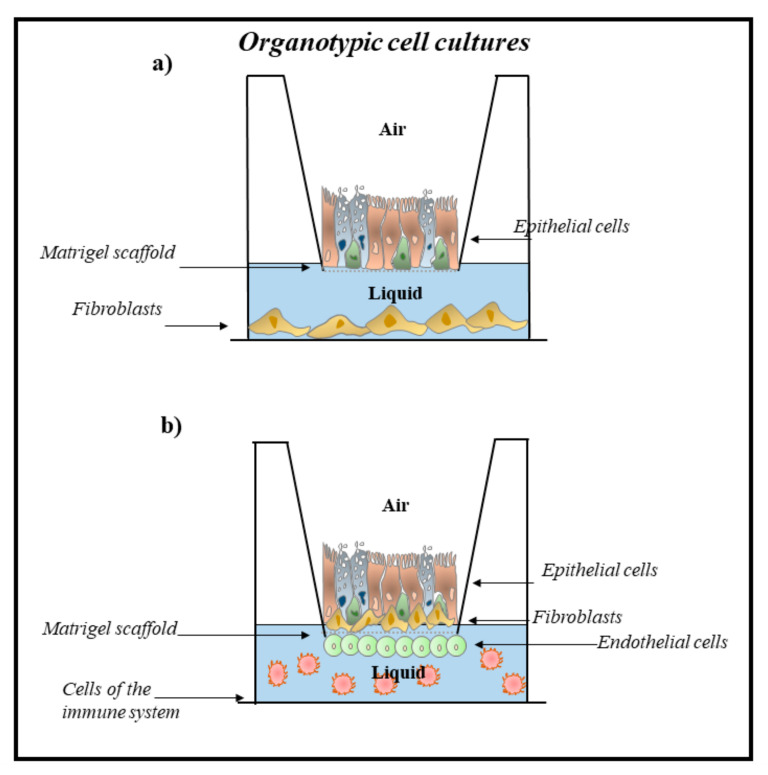
Two different 3D organ-typical cell cultures to study the effects of environmental contaminants in airway diseases of the lung. (**a**) Three-dimensional (3D) cell culture model obtained with a co-culture of epithelial cells and fibroblasts; (**b**) Three-dimensional (3D) cell culture model obtained with multiple cell types from the pulmonary system (structural and inflammatory). Three-dimensional (3D) *tetraculture system* containing macrophages, epithelial cells, fibroblasts, and endothelial cells, mimicking lung organization.

Airway epithelial cells cultured in ALI grow and form a pseudo-stratified epithelium composed of basal, ciliated, and goblet cells typical of a human airway epithelium in vivo [148,149,150,151]. In this culture model, the cells fully differentiate, show tight junctions and cilia, and secrete mucins and protective mediators (e.g., antimicrobial peptides and pro-inflammatory cytokines), representing the in vivo structure and function of the airway epithelium [149]. Thus, the ALI cultures have led to important advances in the characterization of cell biology, in the study of infections, in pharmacological and inhalation toxicity tests of the respiratory epithelium [151,152]. ALI cultures mimic the human airway epithelium and successfully reproduce the lung microenvironment. Furthermore, these cell cultures can punctually reproduce the tissue of origin containing different structural (fibroblasts, endothelial cells, etc.) and inflammatory cell types (macrophages, neutrophils, eosinophils, etc.), and other than polarized epithelial tissue [153]. Currently, there are various 3D organ-typic cell cultures (pulmonary micro-organ) of the airways that aid to understanding many cellular and molecular aspects of the effects of inhaled substances in the lung (Figure 3) [154].

Organ-typic cell cultures or organoids are defined as “cultured structures”. They are built with multiple cell types grouped and spatially organized in a similar way to the organ and show some organ functions [155]. Organ-typic cell models may be obtained with commercially available materials of tissue culture. They are suitable for a variety of experimental projects and for modeling complex lung toxic responses, including inflammation, oxidative stress, myofibroblast formation, transepithelial migration, and invasion. Organ-typical patterns also mimic epithelial barrier properties and changes in cytotoxic and pro-inflammatory effects upon exposure to environmental toxicants such as those observed in vivo [156,157,158].

The term ‘‘organoid’’ defines the 3D culture referring to stem cell-derived native-like tissue structures of a given organ created by the induction of genetically encoded self-assembly programming [159]. Similar to processes that regulate organogenesis during embryonic development, cells within organoids undergo self-organization guided by cell-specific adhesion properties and spatially restricted progenitor differentiation. Organoid culture derived from stem cells or organ-specific progenitor cells that differentiate and self-organize through *cell sorting* and *lineage commitment* similar to the in vivo process. It should be noted that this recent definition is not yet strictly used in the literature [160,161].

**Figure 4:**
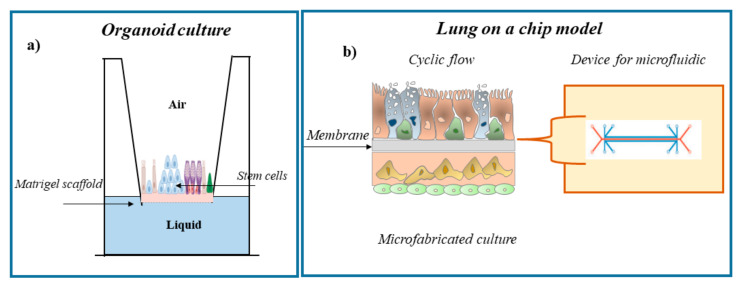
Cell-based in vitro lung models for the study of inhalation toxicity. Visual representation of three-dimensional in vitro models of each model system for the study of inhalation toxicity: (**a**) organoid culture and (**b**) microfluidic and microfabricated device culture. ECM, extracellular matrix.

The gold standard of respiratory system modeling is represented by the lung-on-a-chip (LOC). It is the result of the combined use of most modern techniques of microfluidics and tissue engineering [162,163]. The LOC model provides a microfluidic perfusion system to emulate the cellular microenvironment within the lung with high spatiotemporal precision [162,163]. It is a device made on a transparent glass microplate and made up of three parallel microchannels. The central one is divided into two halves, lower and upper, by a porous membrane, on the two sides of which there are two layers of human cells, respectively endothelial cells of the capillaries and cells of the lining of the lung alveoli. Therefore, half of the channel functions as an airway and half functions as a blood vessel. The two channels are in close adhesion to each other, just as in an in vivo lung. Instead, the two lateral channels have a mechanical function, and when the vacuum is created in the device, they simulate a microdiaphragm [164,165,166]. The LOC system offers new possibilities for inhalation toxicology research. These new technologies may represent an added value in the research; however, these tools are not yet widely available in the scientific community. In fact, technical problems in the use of induced pluripotent stem cells (IPSC) and LOC technology limit their use in the studies of inhalation toxicology [166]. The successful applications of these innovative cell models in pulmonary drug discovery suggests their use in the assessments of inhalation toxicity. Therefore, future research aspires to the validation of innovative models and to the development, optimization, and implementation of new integrated cell models (Figure 4).

Many respiratory diseases begin with the onset of inflammatory reactions that play a key role in pathological lung conditions including asthma, COPD, and interstitial lung disease [63,167]. The airway epithelium is the first line of defense against respiratory lesions from pathogens and toxic agents, as a functional barrier but also to initiate and amplify the immune response [54]. Accordingly, in this review, we have focused our attention on the effects of environmental pollutants on the epithelium of the respiratory tract, and below, we report mainly on some data relating to the simple cell models of 3D ALI cultures.

In recent decades, the exponential use of nanomaterials induces an increased risk of human exposure to nanoparticles (NPs) [122]. Lenz et al. (2013) studied the effect of exposure to zinc oxide (ZnO) NPs present in the air in a double in vitro model of the submerged and ALI culture of human alveolar epithelial cells (A549 cell line) [168]. This study describes that the ALI culture of A549 exposed to ZnO-NPs showed a significant consistent cell response in terms of oxidative stress and inflammation compared to “submerged” cultures of A549. These data together with other references suggest that screening for NP toxicity in vitro with ALI models could produce better results than the results obtained with “submerged” cell cultures [29,30,169,170]. Some studies show that chronic or long-term exposure to gaseous air pollutants may be responsible of long-term respiratory effects such as asthma, allergy, and even the onset of neurological disorders [170]. Formaldehydes, carbon monoxide, and ozone are compounds commonly detected in indoor environments and are responsible for the development of acute toxicity (e.g., respiratory irritation) [170,171]. However, the exposure of the cells to gaseous irritants is obtained with traditional “submerged” in vitro model [172], and the compounds were added to the cell culture medium in liquid form. However, the chemicals added in the medium can alter their properties related to the interactions and binding of components of the medium. This might generate unreliable results [173]. Recently, to test the effects of gaseous compounds on the cells of the airway, the use of an in vitro cell model of ALI cultures is recommended, as they are systems capable of mimicking gas exposure [174]. To date, there are still few commercially available exposure systems that allow studying the effect of gaseous compounds in cell cultures with a precise dosimetry and without any interfering means [175]. Ahmad et al. compare the chlorine toxicity using two epithelial cell models: the “submerged” models and the ALI models [44]. This study shows that chlorine reacts rapidly with aqueous surfaces to form hydrochloric and hypochlorous acid and demonstrates the toxicity of hydrochloric acid rather than chlorine in epithelial cells cultured in submerged conditions [176]. In contrast, the exposure of human airway epithelial cells in differentiated ALI cultures allows a direct interaction between chlorine gas and cell surface in the absence of aqueous media. This type of cell culture is comparable to the realistic exposure scenarios put in place, following the inhalation of gases in environmental and occupational settings.

**Figure 5:**
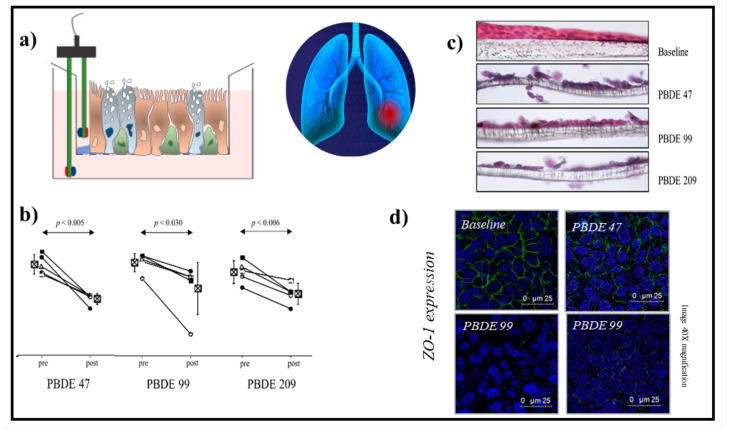
The measurement of TEER in the 3D ALI culture stimulated with air pollutants such as PBDE-47, -99, and -209 modifies the integrity of the epithelium of the lung, promoting airway diseases. (**a**) Electrode useful to measure the values of TEER in 3D cell culture; (**b**) reduction of TEER values before and after the treatment with contaminants of epithelial cells cultured in ALI; (**c**) morphological changes of epithelial cells cultured in ALI; (**d**) the damage of integrity of 3D epithelium is associated with a reduction of ZO-1 expression.

Albano et al. studied the effect of PBDE-47, -99, and -209 using a 3D “in vitro” model of human alveolar epithelial A549 cells (immortalized cell line) or human primary bronchial cells [131] cultured in ALI. In this study, the toxicity of PBDEs was studied with experimental procedures that mimic the exposure of the respiratory epithelium to inhaled contaminants. The data show that PBDE-47, -99, and -209 influence the physiological and biochemical mechanisms of oxidative stress (NOX-4 synthesis), cause inflammation (IL-8 synthesis, changes in the pH of cell fluids), and lead to the mucins’ overproduction and loss of pulmonary epithelial layer integrity measured by the transepithelial electrical resistance (TEER) and the expression of the tight junctions Zonula Occludens-1 (ZO-1). Furthermore, this study provides encouraging evidence to describe a probable effect of the antioxidant N-acetylcysteine (NAC) on some pathological mechanisms generated by exposure to PBDEs in the airway’s epithelial cells. Therefore, the results support the concept that PBDEs could have negative effects on the respiratory epithelium physiology, promoting lung diseases in areas of environmental contamination. The model used in this study represents an important platform for the screening of cell pathogenesis in human airways and turns out to be a powerful tool to improve knowledge of the effects of PBDEs on human health. Indeed, this study highlights that the exposure of airway epithelial cells to PBDEs can generate oxidative stress, inflammation, pH acidification, mucus hypersecretion, increased viscoelasticity property, and loss of epithelial barrier function with subsequent alteration of its integrity (Figure 5).

In fact, these markers of airway disease can be factors involved in the lung function decline, and their levels of the expression in the “in vitro” model of epithelial cells stimulated with PBDEs might suggest the increase of cases of acute and chronic lung disease in the areas of environmental PBDEs contamination (indoor, outdoor). About that, further in vivo studies would be needed to better elucidate the harmful effects of PBDEs on the pathophysiological complex of the airway epithelium as a cause of airway disease and related damage to human health. Therefore, to obtain results of better physiological relevance and understand the role of the airway microenvironment in response to chemicals inhalation, it is necessary to develop organ-typical (micro-organ) models capable of modeling 3D lung microenvironments using different type of lung cells organized in co- or multicultural systems.

## 5. Exposome, Omics Technologies, and New Biomarkers (miRNA) in Toxicology

The modern concept of the exposome constitutes a new paradigm for studying the impact of external agents and various substances on gene expression to evaluate the consequences and the effects on human health. Evidence shows how the genetic predisposition of each subject adds up to environmental exposure in the onset of chronic diseases [177]. Recently, an alternative definition of exposure has been proposed that explicitly incorporates hereditary risk factors, the response of the body to environmental pollution, and endogenous metabolic processes that can alter human biochemical mechanisms [178].

Toxic-epigenomics allows us to study the effects of individual contaminants on the dysregulation of gene expression; the most used for studies on the subject are organic and non-organic compounds, which are found by analyzing air, water, and soil. Omics technologies are a promising technique for integrating a wide range of environmental exposures to a small number of biological matrices. This term is associated with different fields of biology, generally genomics, transcriptomics, proteomics, metabolomics. All these data, analyzed together, allow a broader understanding of the individual subject’s response to environmental exposure. Recent data show that the omics technique contributes to identify specific biomarkers involved in the causal relationship between PM_2.5_ pollution and deleterious lung outcomes [179].

However, this type of approach is still particularly difficult, especially due to its high cost. Methylome has been of great interest for some time. In fact, epigenetic modifications remain of great interest in the scientific community; in fact, the study of a cohort of subjects exposed to different types of pollutants and chemicals demonstrates how there is a variation in DNA methylation performed by gene sequencing [180].

The presence of altered levels of molecules at the systemic level can indicate the progress of specific diseases, which is currently of great interest in the scientific community. In recent years, RNA molecules have been the subject of great interest, and these studies led to the identification of microRNAs. MicroRNAs are sequences of approximately 22/25 nucleotides; they are transcribed from non-coding regions of the genome, undergo post-transcriptional changes that lead them to “ripening”, and at that point, they are ready to perform their function [181]. Their mechanism of action involves the binding of a complementary portion of a mature mRNA to regulate its function: blocking its translation or leading to degradation [177]. Some microRNAs regulate dozens of targets RNAs ensuring the maintenance of physiological processes in all tissue districts: cell proliferation, differentiation, balancing of the oxidative stress, metabolism, and apoptosis [182]. Alterations in the expression of these microRNAs lead the cell to lose its homeostasis [183]. Computational biology gives us a great help in the prediction of the target of the microRNA. Through specific databases, the gene sequences are analyzed to identify predictive targets of the microRNAs examined (the homologous nucleotide sequences of the microRNAs that are complementary and that can potentially be regulated by the microRNA under consideration) [184].

External factors, and in particular environmental pollutants, play a key role in the perturbability of cell functions such as the alteration in the expression of regulatory microRNAs and the consequent gene expression [185]. In the context of human health, recent studies have associated the increase or decrease of some microRNAs in relation to certain diseases or to simple exposure to cigarette smoke [186]; generally, they are clusters made up of some tens of microRNAs that see their expression jointly altered; epigenetic alterations and changes in regulatory pathways also lead to connections between inflammatory mechanisms and cancer [185,187].

Polychlorinated biphenyls (PCBs) have been widely used over the years, and their production has recently been banned due to their environmental impact; several studies have shown how their bioaccumulation in fatty tissues causes a deregulation in gene and microRNA expression [188]. Arsenic in its trivalent form (As[3]^+^) is also associated with cancer risk, and environmental exposure to arsenic, especially long-term, is associated with gene instability and the risk of diseases associated with peripheral vascular lesions [181]. High concentrations of mercury (Hg) in the blood are related to the risk of hypertension, and in general of cardiovascular toxicity, endothelial toxicity, hypercholesterolemia, and neurotoxicity, especially when exposure to this heavy metal occurs during prenatal development [189]. HUVEC cell lines were used to demonstrate the effects of Hg on the expression of miR-92a and miR-486; the parallel analysis of the plasma of workers exposed to Hg confirms the increase in the expression of the same microRNAs. This demonstrates how through integrated approaches of in vitro/in vivo studies, it is possible to demonstrate an aberrant alteration of molecular biomarkers that interfere in the activation pathways of inflammatory mechanisms involving NF-κB and the expression of COX-2 altering the physiological apoptotic processes [190].

The perfect biomarkers should be readily available in the body fluids, such as blood, liquid biopsies, or others. Furthermore, it should also be tissue or cell-specific type and undergo a variation in the expression to be also useful for the control and response to the therapy used [191]. So, to identify specific biomarkers of diseases, it is important that an initial approach includes in vitro studies. This approach provides us tools to study and analyze the biosensors of one cell type at a time. It is highly repeatable, reproducible, and gives the opportunity to connect the levels of biosensor detection of expression directly to the stimulus used in the experimental procedures [192]. Finally, an integrated approach between the three experimental models enclosing in vitro, ex vivo, and in vivo studies allows us to study quickly the direct effects of the molecules of interest involved in the environmental contamination due to toxicants such as PBDEs. However, the validation of biomarkers as biosensors passes from the analysis of biological samples and possibly from the effect of a potential drug that restores or suppresses its expression and activity.

The bronchial epithelium constitutes the first barrier to the inhaled pollutants present in the environment; the increase in oxidative stress as well as the release of pro-inflammatory cytokines and exosomes containing microRNA is now widely demonstrated both by in vitro and in vivo studies [193]. Multi-organ studies, ex vivo, give us indications on molecular biomarkers related to inflammatory processes of the airways such as the Let-7, mir21, mir122, and mir25 microRNA families [181].

PBDE induce a variation in the expression of biomarkers related to inflammatory processes of the lung in an in vitro/ex vivo study performed on human lung epithelium cells (epithelial cell line and cells isolated from lung biopsies). We studied the effects of three different PBDEs (47, 99, and 209) in the mechanisms of oxidative stress, epithelial integrity, and release of inflammatory cytokines in the cell culture of bronchial epithelial cells [130,131]. Furthermore, the analysis of the transcripts allowed us to isolate the microRNAs secreted both by immortalized epithelial cells (A549) and primary human bronchial epithelial cells. We highlighted a decrease in the expression of let-7a together with an increase of both mir21 and mir25 microRNA [193]. These results support the concept that biomarkers detected in the in vitro model of cell culture might be a useful tool in the prediction of lung diseases and their progression in subjects exposed in risk areas.

Placenta is fundamental in the regulation of the intrauterine environment, and it is already known that heavy metals such as Cd and lead together with other environmental pollutants can modify the expression of various microRNAs [190]. To support our in vitro/ex vivo approach with a 3D cell culture of epithelial cells [193,194], where we highlighted the let-7a, mirR21, and mirR25 microRNAs production as molecular biomarkers of PBDE contamination, in future studies, we will analyze a cohort of pregnant women exposed to PBDEs present in the environment as pollutants.

**Figure 6:**
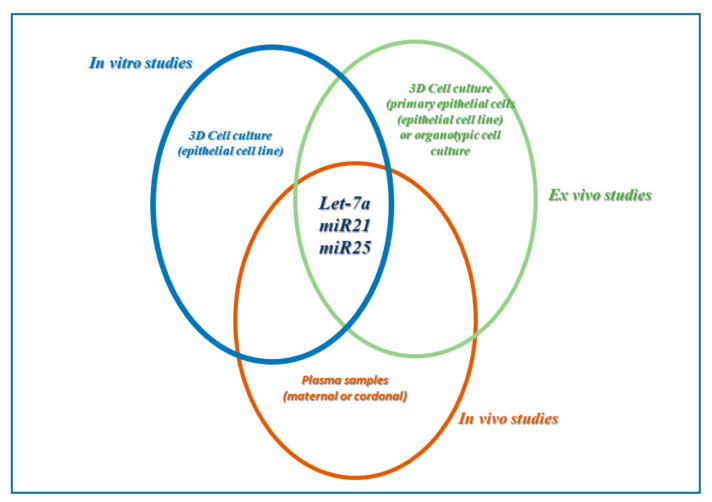
Experimental approach to identify molecular biosensors of airway disease. “In vitro/ex vivo” studies obtained with experimental approach performed with 3D cell cultures of bronchial epithelial cells (cell line and primary cells); Integrated approach obtained including “in vitro”, ex vivo” 3D cell cultures of bronchial epithelial cells and “in vivo” studies.

The integrated approach between in vitro ex vivo and in vivo studies proves to be the key to identifying the patterns of molecular biomarkers (miRNA) that can help in the early diagnosis of organ pathology and facilitate the identification of the response to drug treatment, to use a personalized therapy tailored to the patient exposed in the areas of environmental contamination (Figure 6).

## 6. Conclusions

Environmental contamination plays a fundamental role in human health, generating serious diseases such as inflammatory and neoplastic ones. In this review, we described some effects of air environmental contamination on human health, regarding lung diseases. We realized a descriptive approach with the aim to transfer the following in a simple way: (1) fundamental aspects of the activation of epithelial cells in respiratory diseases in case of exposure to contamination environmental; and (2) useful tools for an adequate in vitro/ex vivo experimental design to study the effect of air pollutants in the lung. About this last point, we particularly refer to our experiences regarding the use of 3D ALI cultures of epithelial cells.

In this scenario, novel 3D in vitro models offer the advantage of enhanced physiological relevance through the incorporation of architectural support (i.e., ECM proteins or scaffolding), cell–cell interactions, and in some instances, biomimetic devices that can recapitulate physiologically breathing motions. However, despite their contributions, cell models have not been able to accurately represent the heterogeneity of the human population and account for interindividual variability in response to inhaled toxicants and susceptibility to the adverse health effects.

In this review, we focalized our attention to the concerns about the effects of air environmental contamination on human health regarding diseases of the lung with particular attention to the role of epithelial cells. Our descriptive approach has been discreet with the aim to transfer in a simple way the fundamental aspects of the activation of epithelial cells in respiratory diseases in case of exposure to environmental contamination. Here, we refer to our experiences about the use of 3D ALI cultures of epithelial cells to study the effect of some toxicants on epithelial cells.

Furthermore, here, we introduced the concept of the exposome, since it constitutes a new paradigm for studying the impact of the environment on human health. Finally, the contribution of *Omics Sciences* defined new scientific perspectives aimed at the discovery of the cellular and molecular mechanisms underlying the immunological response of the airway epithelium in conditions of environmental air contamination. The goal of the researchers might be to enrich the concept of exposome using innovative biological systems that mimic organ situations in real life.

In conclusion, with the short descriptions enclosed in this review, we underline and suggest the importance of planning new technologic and conceptual perspectives useful to further clarify the effect of environmental factors on the health of the lung. The specific objectives to be achieved are to identify new cellular and molecular pathways associated with the concept of the exposome, with the help of complex organotypic and organoid cultures with applications of microfluidics and omics sciences.

## Abbreviations

FVC	forced volume capacity
COPD	chronic obstructive pulmonary disease
CO	carbon monoxide
NOx	nitrogen oxides
SO_2_	sulfur dioxide
IPA	polycyclic aromatic hydrocarbons
PM	particulate matter
VOC	volatile organic compounds
COHb	carboxyhemoglobin
NO2	nitrogen dioxide
B[a]P	benzo-[a]-pyrene
IARC	International Agency for Research on Cancer
ESCAPE	European study of cohorts for air pollution effects
SIN	sites of national interest
CISAS	International Center for Advanced Studies in Environment, Ecosystem and Human Health
OMS	World Health Organization
POP	persistent organic pollutants
PCB	polychlorinated biphenyls
PBDE	polybrominated diphenyl ethers
EFSA	European Food Safety Authority
TLR4/NF-kB	Toll-like receptor 4/nuclear factor kappa-light-chain-enhancer of activated B cells
UE	European Union
AM	alveolar macrophages
TNF-α	tumor necrosis factor
IL-	interleukin-
DAMP	damage-associated molecular pattern
GM-CSF	granulocyte-macrophage colony-stimulating factor
MCP-1	monocyte chemoattractant protein 1
TSLP	timic stromal lymphopoietin
O_3_	ozone
C_6_H_6_	benzene
As	arsenic
Cd	cadmium
Ni	nickel
ROS	reactive oxygen species
DUOX1	duox ossidasi 1
NADPH	nicotinamide adenine dinucleotide phosphate
Nrf2	erythroid 2-like 2
ALI	air–liquid interface
LOC	lung-on-a-chip
IPSC	induced pluripotent stem cells
NP	nanoparticles
TEER	transepithelial electrical resistance
NAC	N-acetyl cysteine
Hg	mercury
COX-2	cyclooxygenase-2
RNA	ribonucleic acid
mRNA	messenger RNA
Zonula Occludens-1	ZO-1
